# Teleneuropsychology: normative data for the assessment of memory in online settings

**DOI:** 10.1007/s10072-022-06426-9

**Published:** 2022-10-05

**Authors:** Ezia Rizzi, Michela Vezzoli, Sara Pegoraro, Alessio Facchin, Veronica Strina, Roberta Daini

**Affiliations:** 1 Department of Social and Human Science, University of Salento, Studium 2000, Via di Valesio, 73100 Lecce, Italy; 2 Department of Psychology, University of Milano-Bicocca, Milan, Italy

**Keywords:** Online setting, Teleneuropsychology, Normative data, Digit Span, Rey Auditory Verbal Learning, Verbal Paired Associated Learning

## Abstract

**Introduction:**

The COVID-19 pandemic has forced significant changes in clinical practice. Psychologists and neuropsychologists had to modify their settings to assess patients’ abilities, switching from an in-person modality to a remote setting by using video calling platforms. Consequently, this change brought about the need for new normative data tailored to remote settings.

**Aim and methods:**

The study aimed to develop normative data for the online assessment of neuropsychological memory tests and to compare it with the published norms obtained in standard settings. Two hundred and four healthy Italian volunteers performed three verbal memory tests through the Google Meet platform: the Digit Span (Backward and Forward), the Rey Auditory Verbal Learning, and the Verbal Paired Associated Learning Test.

**Results:**

This research provides specific norms that consider the influence of demographic characteristics. Their comparison with published norms shows a medium to high agreement between systems. The present study provides a reference for the clinical use of neuropsychological instruments to assess verbal memory in a remote setting and offers specific recommendations.

**Supplementary Information:**

The online version contains supplementary material available at 10.1007/s10072-022-06426-9.

## Introduction

The COVID-19 pandemic has prompted governments worldwide to establish mandatory rules to avoid infection, such as using personal protective equipment (e.g., wearing a mask) and social distancing. Emergency has led to several changes in our daily life and work habits. In this context, neuropsychologists have resorted to alternative methods to conduct assessments, rehabilitation, and cognitive stimulation in clinical and research contexts. Specifically, they have had to reorganise clinical settings by switching from face-to-face to remote communication via videoconferencing. Previous research has shown that online assessment is a reliable alternative for diagnosing neurocognitive and degenerative disorders [1, 2], such as mild cognitive impairment (MCI), preclinical dementia, as well as in healthy population assessment [3] and Alzheimer’s disease [4]. According to a pre-pandemic study of American Indians living in rural areas, some patients with cognitive impairments and their families preferred remote neuropsychological assessment over face-to-face assessment [5]. In addition, another study carried out on American Indians as well, demonstrated the remote setting feasibility and reliability [6]. Situational and personal factors influence patients’ preference for remote settings. Several studies have demonstrated that the elderly prefer the remote setting, especially if they live in isolated areas with insufficient health care resources [7], suffer of physical and motor limitations, and experience economic constraints [8–10].

Similarly, studies involving veterans troubled by dementia found that they were more satisfied with the possibility of performing a neuropsychological assessment by video conference rather than travelling to the clinic themselves [11, 12]. On the whole, studies demonstrated many advantages of videoconferencing in clinical practice: it makes it easier for patients to access medical care and permits saving essential resources (e.g., time and money); patients live it as a positive experience, as also demonstrated in other medical services and care [6, 13]. Lastly, a remote clinical setting can help evaluate people suffering from interpersonal anxiety, which negatively affects the performance of neuropsychological tests [8, 14]. From a life-span perspective, videoconferencing could be considered an additional neuropsychological assessment service that can improve the diagnosis and permit better supervision of cognitive disorders over time of patients with various neurological and other medical conditions [15]. Despite the many advantages of videoconferencing in neuropsychological assessment, it also has its limitations which might compromise the validity and reliability of the assessment. Remote assessments rely entirely on technological devices (e.g., laptops, tablets, smartphones) that, however, might malfunction during the session. Moreover, video and audio quality variability during videoconferencing (e.g., transmission delays) can invalidate tasks that require time/speed recording [16].

In the case of visual tests, differences in device settings (e.g., video size and resolution) may cause problems. For example, when doing visuoconstructional tasks with smartphones, patients may unintentionally miss parts of the figure or enlarge/alter it. Also, distractions in patients’ environments can invalidate the assessment. Patients’ technology skills are also crucial for the validity of the online evaluation [17], as the more they interact with technological devices, the more it improves the quality of the session and, thus, the assessment. Furthermore, compared to traditional settings, where the physical proximity to patients permits neuropsychologists to keep more control over their behaviour (e.g., note-taking correct answers during the assessment), in remote sessions, it might be hard to control patients’ behaviour to the same extent. The validity of the evaluations is compromised when these behaviours are difficult to control [18]. In the final analysis, studies demonstrated that many neuropsychological tests in the remote setting obtained similar scores to face-to-face testing and no differences in participants’ performance [19, 20]; therefore, they can be considered comparable. Analysing in detail, most of these studies adapted verbal task tests, such as the Digits Span Forward and Backward, the Boston Naming Test [6, 20], and the Hopkins Verbal Learning Test [18] in adults and senior adults. In a study [21] carried out in a French memory clinic, patients performed a neuropsychological evaluation two times (i.e., face-to-face and teleneuropsychology). The second assessment took place 4 months later, adopting parallel versions of neuropsychological tests. During the teleneuropsychological assessment, patients were in a room with an assistant in case they needed to use the device. The neuropsychological evaluation included tests of Verbal Fluency, Digit Span, and Free and Cued Selective Reminding Test (FCSRT). In addition, the authors evaluated patients’ satisfaction with the videoconferencing method via a five-point Likert scale, their anxiety during the tele-evaluation, and their preference between the two settings. The results endorse the feasibility and reliability of teleneuropsychology. Due to the considerable use of videoconferencing platforms to limit the emergence of problems during remote assessment, the National Board of Italian Psychologists published an informative summary on how to conduct a neuropsychological assessment remotely. According to this document, tests must be administered in a quiet and isolated place to ensure patient privacy and avoid distractions; the room light should be adequate, and wearing earphones is recommended. In the course of the session, to limit the possibility of interruptions, it is crucial to have a stable Internet connection: an Ethernet cable is favoured over a Wi-Fi connection. In this context, however, a gap remains evident: most previous studies do not consider the need to provide regulatory data suitable for administering online tests. Focusing on the advantages or disadvantages of online administration or providing recommendations for improving the setting are fundamental aspects; nevertheless, it is crucial also to develop normative data to guide the interpretation of the performances evaluated at a distance. Non-standardised evaluation procedures can lead to mistakes in interpreting scores and global performance evaluation. It is necessary to outline a quality methodology to obtain a reliable and accurate cognitive evaluation and recommendations for administration [8, 22, 23]. Therefore, before generalising procedure and application, there is a requirement for new standardised measures and normative data, in healthy or patients, with the same validity of normative data employed in a classic setting [21]. In fact, the lack of normative data could cause diagnostic errors and adversely affect health and access to treatment [22, 24]. The studies mentioned above have already used verbal memory tests in a remote setting, especially for specific categories of the population and for specific ages (i.e., dementia and adults), demonstrating that they can be employed with results comparable to those in a face-to-face setting, and confirming the validity of such administration. Recently, some cognitive and behavioural screening tests were standardised for application in telehealth care in the Italian population. The aim was to provide telephone-based first-level assessments and evaluations of frontal abilities: Telephone Interview for Cognitive Status [25], Telephone-based Frontal Assessment Battery [26], and ALS Cognitive Behavioural Screen-Phone Version [27]. In a recent review, telephone, videoconference, and web-based brief screening instruments for cognitive and behavioural impairment have demonstrated the validity, reliability, and usability in tele-neuropsychology [28]. In light of previous studies, and considering the wide use of memory tests in diagnosis (e.g., MCI, Alzheimer’s), our research aims to fill the gap in the psychometric and neuropsychological fields by providing normative data specific for videoconference measurement of short-term memory, working memory, and long-term memory (i.e., Digit Span Forward and Backward, Rey’s Auditory Verbal Learning Test, and Verbal Paired Associated Learning Test) and comparing results with standardised norms used in face-to-face settings. Also, this research aims to provide practical suggestions and tricks for online testing.

## Materials and methods

### Participants

The study includes 207 participants (i.e., Italian volunteers, classified as students, workers, and unemployed) (see Table S1 in Supplementary Materials), recruited through flyers, social media, and mailing lists of previous unrelated studies. Data were collected in 2021 during the second wave of the COVID-19 pandemic. A sensitivity power analysis with G*Power [29] was conducted to assess the minimum effect size that we can validly observe with the available sample size (*N* = 204), *α* = 0.05 and 1-*β* = 0.80 in a multivariate regression model with three predictors (i.e., age, gender, and education). The results indicate that a small effect (*f*^2^ = 0.038) [30] is detectable with the given parameters. Volunteers did not receive any fees or gifts for their participation. All participants identified themselves as female or male. Therefore, the sample consists of 204 participants (127 females) mean age of 44.33 years (SD = 18.52, range 18–84) and a mean education of 15 years (SD = 3.85, range 5–26). Participants were included following specific inclusion criteria: no previous history and actual neurological or psychiatric disorders, a corrected score above 19.5 on the Montreal Cognitive Assessment (MoCA) [31], and no visual and hearing impairments (i.e., maculopathy and partially-*sighted*). Participants performed the MoCA in the same session, before memory tests. Also, participants who avoided looking directly at the camera or who were suspected of note-taking correct answers were excluded from the analysis (*n* = 3). We collected 199 valid answers on the Digit Span Forward and Backward (mean age = 43.62, SD = 18.19, range 18–84, mean education = 15.1, SD = 3.8, range 5–26, 123 females), 180 on Rey’s Auditory Verbal Learning Test (mean age = 40.80, SD = 16.38, range 18–80, mean education = 15.46, SD = 3.5, range 5–26, 108 females), and 204 volunteers on the Verbal Paired Associates Learning. Participants had a chance to decide freely which test took part in. A sensitivity power analysis (G*Power [29]) for a multivariate regression model with three predictors showed that we were still able to detect a small effect size (*f*^2^ = 0.044) with a sample of 180 participants (i.e., the minimum sample size we obtained), *α* = 0.05 and 1-*β* = 0.80. Participants provided their informed consent before starting the study using Google modules. The Ethical Committee of the University of Milano Bicocca approved the study (Protocol n. RM-2020–360) and complied with the Declaration of Helsinki.

### Procedure

Neuropsychological tests were administered using Google Meet, a videoconferencing application that does not require specific apps for laptops and allows easy access to tablet apps. Data collection and storage through Google services are compliant to the Health Insurance Portability and Accountability Act (HIPAA), a law enacted to regulate the use, disclosure, and protection of protected health data, following the digitalisation of all health-related information. Examiners used Asus VivoBook 15 or MacBook Air. Participants sat in front of their personal computers or tablet while wearing headphones and listened to the experimenter’s instructions. The examiner was in the university room, and participants were at their place via remote support. They had to look directly into the computer camera and keep their hands close to their chins to prevent note-taking. We asked them to remain alone in the room and turn off devices that could distract them (e.g., smartphones, TV, radio). To reduce interference between Rey’s Auditory Verbal Learning Test and Verbal Paired Associates Learning, participants were asked to copy seven draws of the Constructional Apraxia Test [32] and Raven’s Progressive Matrices (CPM) [33] between Rey’s Auditory Verbal Learning Test immediate and delay recall. The present research elaborated normative data of three neuropsychological verbal memory tests. Digit Span Forward (DSF) and Backward (DSB) measured verbal short-term and working memory. The examiner read a series of digits (i.e., numbers), and the participants had to repeat them in the same order (forward span) or reverse order (backward span). The experimenter proposed a more extended sequence of digits if participants correctly recalled the previous one. The total scores were the most extended Digit Span correctly recalled in each subtest, forward (DSF) and backward (DSB) (ranging from 0 to 9) [34]. The second test, Rey’s Auditory Verbal Learning Test, assessed retrograde and anterograde memory (i.e., immediate recall, RAVL-I, and delayed recall, RAVL-D) [35]. The examiner read a verbal list of 15 words, and the participants recalled as many words as possible. The procedure was repeated five times, and the responses were counted as RAVL-I scores (ranging from 0 to 75). Fifteen minutes later, the participants recalled previously learned words (RAVL-D, scores ranging from 0 to 15). The scores of the RAVL-I and RAVL-D were the highest number of correctly recalled words. Verbal Paired Associated Learning (VPAL) test was also administered to evaluate anterograde memory and learning. The examiner read ten pairs of words (e.g., fruit-grape). After presenting the whole list, the participants listened to one of the words learned before and had to repeat the second (e.g., the examiner said fruit, and the participants said the word paired, thus grape). The procedure was repeated three times, and the order of the words in the list changed every time. The word pairs list contained five easier pairs and five complex pairs. One point was awarded for each more complicated pair and half a point for each easy pair. The total score was the highest number of word pairs recalled correctly (scores ranging from 0 to 22.5) [36]. The participants performed tasks in an approximately 45-min virtual individualised single session and in the same order (i.e., Rey’s Auditory Verbal Learning Test Immediate, Rey’s Auditory Verbal Learning Test, Rey’s Auditory Verbal Learning Test Delayed Recall, Constructional Apraxia Test, Verbal Paired Associates Learning, and Digit Span Forward and Backward).

### Statistical analyses

To test for normality assumption, we evaluated skewness and kurtosis for sociodemographic and tests scores. Skewness and kurtosis were judged as abnormal if their value exceeded |1| and |3|, respectively [37]. Table 1 presents the descriptive statistics of the measured variables and their correlations. Correlations between continuous variables were estimated through Pearson’s *r* correlation, whereas correlations between continuous variables and the dichotomous variable (i.e., gender) were estimated through point-biserial correlation.

**Table 1:** Sample descriptives and correlations between variables

Variable		*M* (*SD*)/*N*	Range	1	2	3	4	5	6	7
1	Gender (m/f)	77/127								
2	Age	44.33 (18.51)	18–84	.06						
3	Education	14.98 (3.85)	5–26	.00	− .49***					
4	DSF	6.40 (1.15)	4–9	− .21***	− .21***	.14				
5	DSB	5.01 (1.26)	2–8	− .12	− .29***	.22***	.52***			
6	RAVL-I	54.09 (9.12)	19–71	.24 ***	− .46***	.39***	.28***	.31***		
7	RAVL-D	11.82 (2.65)	3–15	.32***	− .41***	.38***	.21***	.28***	.78***	
8	VPAL	16.60 (4.25)	3–23.5	.10	− .55***	.40***	.32***	.32***	.65***	.57***

*DSF*, Digit Span Forward; *DSB*, Digit Span Backward; *RAVL-I*, Rey’s Auditory Verbal Learning Test-Immediate; *RAVL-D*, Rey’s Auditory Verbal Learning Test-Delayed; *VPAL*, Verbal Paired Associates Learning Test. *p < .05; **p < .01; *** *p* < .001

To define normative values, we used a regression-based procedure that tests, analyses, and removes confounding effects of demographic factors [38]. Also, this procedure permits obtaining valid normative data using a sample in the order of hundred participants [38]. For each test, bivariate regression analysis was initially conducted to define the best transformation of the sociodemographic variables. Subsequently, the significant predictive factors were entered in a stepwise regression to identify which of them best predict a test score in a multivariate context. The most predictive factors (in their best transformation) and the dependent variable were then transformed by calculating their deviations from the sample mean. The obtained variables were finally introduced into a new multivariate regression model, and the resulting coefficients were reversed in sign to remove significant variation in the outcome explained by the sociodemographic predictors. Adjusted scores were computed based on this latter equation.

The Equivalent Scores (ES) [38] method was used to score normative values from adjusted test scores. First, we identified the internal (ITL) and external (OTL) 95% non-parametric tolerance limits with 95% CI. In accordance with their definition, ES = 0 was defined by the OTL, ES = 4 by the median, and intermediate ESs (i.e., ES 1, 2, and 3) were defined using a rank-based approach [39]. In addition to the ESs, percentile scores were calculated.

In order to explore and compare the diagnostic performance of the normative data thus obtained to the norms developed in traditional settings and reported in the literature, we performed a simulation analysis based on a simulated dataset of amnestic Mild Cognitive Impairment (aMCI) patients. We chose an aMCI population for the comparison because their assessment usually involves the same memory tasks used in our study, and there are published Italian data available [40, 41]. The simulated data, based on the published means and standard deviations of the five target test scores, allowed us to carry out the analysis even if we could not recruit actual aMCI patients. We simulated data from 100 participants. Due to a well-documented correlation between sociodemographic variables, age and education were assigned pseudo-randomly to allow them to correlate negatively (*r* =  − 0.22, *p* < 0.05). The simulation analysis permitted us to overcome the issues of descriptively compare different normative scoring systems. Firstly, the two normative diagnostic systems rely on different adjustment regression equations that were derived from data collected in different settings (i.e., online vs face-to-face) and times (1990s vs 2021). Secondly, to reliably compare the classification ability of different normative systems, one must use a sample independent to the one used to develop the normative system itself. That is, it was essential to determine whether the model’s predictions generalise well to unseen data. Furthermore, this sample should reflect the characteristics of a clinical sample, because the performance of a classification system on a healthy sample could be biassed due to class imbalance (i.e., the number of healthy cases outweighs the number of pathological cases). To compare the diagnostic performance of normative scoring systems, we assessed classification accuracy and area under the curve (AUC). To further assess the agreement between norms, the Cohen’s Kappa and AC1 indexes were computed. The latter was used to overcome the Kappa difficulties in dealing with class imbalance [42], which manifests when one class (i.e., impaired individuals) is far more numerous than another (i.e., healthy individuals). The alpha level was set to 0.05. All analyses were carried out using the R statistical environment [43]. Supplementary materials are available at https://osf.io/6wra7

## Results

In general, participants did not show any difficulties in performing the tasks remotely. They did not require assistance from a caregiver during the session. Also, all the participants and the experimenter did not experience Internet connection failures or problems with the devices used.

### Descriptive statistics

Descriptive analysis revealed that all variables were normal: skewness values ranged from 0.17 to 0.97; whereas kurtosis values ranged from 0.17 to 1.20.

As shown in Table 1, cognitive scores were significantly correlated with each other, ranging from small (DSF and RAVL-D, *r* = 0.21, *p* < 0.001) to very large (RAVL-I and RAVL-D, *r* = 0.78, *p* < 0.001) values. Gender was negatively associated with DSF (*r*_*pb*_ =  − 0.21, *p* < 0.001) and positively with the RAVL-I (*r*_*pb*_ = 0.24, *p* < 0.001) and RAVL-D (*r*_*pb*_ = 0.32, *p* < 0.001). In other words, female participants showed a worse performance than male on the DSF and a better performance on the RAVL-I and RAVL-D. All cognitive scores were negatively associated with age using correlation values ranging from small to large (− 0.21 ≤ *r* ≤  − *0.5*5,* p* < 0.001). Education correlated significantly with all cognitive scores (0.22 ≤ 0.40, *p* < 0.001), but only marginally with the DSF (*r* = 0.14, *p* = 0.056).

### Normative data definition

The results of stepwise multivariate regressions show how the sociodemographic factors (i.e., gender, age, and education level) predict test scores following different patterns. While the scores of the RAVL-I, RAVL-D, and VPAL were significantly predicted by all sociodemographic variables (all *p* < 0.026), DSF scores were significantly predicted by age (*p* = 0.003) and gender (*p* = 0.004), and DSB scores only by participant’s age (*p* < 0.001). In Table S2 (see Supplementary Materials), we reported the adjusted regression equations for all cognitive scores along with the three metrics (i.e., *R*^2^, adjusted *R*^2^, and the Root Mean Square Error) employed to evaluate model predictive performance. These equations were employed to adjust raw scores. To facilitate the clinical adjustments of raw scores, we computed the adjustment grid for each test (see Table 2). Using the adjusted scores, we calculated the OTL and ITL (see Table S3 in Supplementary Materials), necessary for determining the ESs. Following the procedure described in [39], we computed the ESs cut-offs and reported them in Table 3. Finally, percentile scores were calculated and reported in Table 4.

**Table 2:** Correction grid for computing the adjusted score

Test		Age														
		18	22	27	32	37	42	47	52	57	62	67	72	77	82	84
DSF																
Female		− 0.03	− 0.02	− 0.01	0.02	0.05	0.09	0.14	0.20	0.28	0.37	0.47	0.6	0.74	0.9	0.97
Male		− 0.50	− 0.49	− 0.48	− 0.46	− 0.42	− 0.38	− 0.33	− 0.27	− 0.2	− 0.11	0.00	0.12	0.27	0.43	0.5
DSB		− 0.51	− 0.43	− 0.33	− 0.23	− 0.13	− 0.03	0.07	0.17	0.27	0.37	0.47	0.57	0.67	0.77	0.81
RAVL-I																
Female	5	8.91	9.31	9.84	10.41	11.04	11.73	12.51	13.38	14.39	15.57	17.00	18.83	21.35	25.42	28.15
	8	0.96	1.35	1.88	2.46	3.09	3.78	4.55	5.43	6.43	7.61	9.05	10.88	13.39	17.47	20.19
	13	− 4.14	− 3.75	− 3.22	− 2.64	− 2.01	− 1.32	− 0.55	0.33	1.33	2.51	3.95	5.78	8.29	12.37	15.09
	16	− 5.67	− 5.28	− 4.75	− 4.17	− 3.54	− 2.85	− 2.08	− 1.20	− 0.20	0.98	2.42	4.25	6.76	10.84	13.56
	18	− 6.41	− 6.01	− 5.48	− 4.91	− 4.28	− 3.59	− 2.81	− 1.94	− 0.93	0.25	1.68	3.51	6.03	10.1	12.83
	21	− 7.25	− 6.85	− 6.32	− 5.75	− 5.12	− 4.43	− 3.66	− 2.78	− 1.78	− 0.59	0.84	2.67	5.19	9.26	11.98
Male	5	13.13	13.52	14.05	14.63	15.25	15.95	16.72	17.59	18.60	19.78	21.22	23.04	25.56	29.64	32.36
	8	5.17	5.57	6.10	6.67	7.30	7.99	8.77	9.64	10.64	11.83	13.26	15.09	17.61	21.68	24.41
	13	0.07	0.47	1.00	1.57	2.20	2.89	3.67	4.54	5.55	6.73	8.16	9.99	12.51	16.58	19.31
	16	− 1.46	− 1.06	− 0.53	0.04	0.67	1.36	2.14	3.01	4.02	5.20	6.63	8.46	10.98	15.05	17.78
	18	− 2.19	− 1.8	− 1.27	− 0.69	− 0.07	0.63	1.40	2.27	3.28	4.46	5.9	7.72	10.24	14.32	17.04
	21	− 3.04	− 2.64	− 2.11	− 1.54	− 0.91	− 0.21	0.56	1.43	2.44	3.62	5.05	6.88	9.40	13.47	16.20
RAVL-D																
Female	5	1.99	2.18	2.42	2.66	2.90	3.14	3.38	3.62	3.86	4.10	4.34	4.58	4.82	5.06	5.16
	8	− 0.16	0.03	0.27	0.51	0.75	0.99	1.23	1.47	1.71	1.95	2.19	2.43	2.67	2.91	3.01
	13	− 1.54	− 1.35	− 1.11	− 0.87	− 0.63	− 0.39	− 0.15	0.09	0.33	0.57	0.81	1.05	1.29	1.53	1.63
	16	− 1.96	− 1.76	− 1.52	− 1.28	− 1.04	− 0.8	− 0.56	− 0.32	− 0.08	0.16	0.40	0.64	0.88	1.12	1.22
	18	− 2.16	− 1.96	− 1.72	− 1.48	− 1.24	− 1.00	− 0.76	− 0.52	− 0.28	− 0.04	0.20	0.44	0.68	0.92	1.02
	21	− 2.38	− 2.19	− 1.95	− 1.71	− 1.47	− 1.23	− 0.99	− 0.75	− 0.51	− 0.27	− 0.03	0.21	0.45	0.69	0.79
Male	5	3.62	3.81	4.05	4.29	4.53	4.78	5.02	5.26	5.50	5.74	5.98	6.22	6.46	6.70	6.80
	8	1.47	1.66	1.90	2.14	2.38	2.62	2.87	3.11	3.35	3.59	3.83	4.07	4.31	4.55	4.64
	13	0.09	0.28	0.52	0.76	1.01	1.25	1.49	1.73	1.97	2.21	2.45	2.69	2.93	3.17	3.27
	16	− 0.32	− 0.13	0.11	0.35	0.59	0.83	1.07	1.31	1.55	1.79	2.03	2.28	2.52	2.76	2.85
	18	− 0.52	− 0.33	− 0.09	0.15	0.39	0.63	0.87	1.11	1.35	1.60	1.84	2.08	2.32	2.56	2.65
	21	− 0.75	− 0.56	− 0.32	− 0.08	0.16	0.41	0.65	0.89	1.13	1.37	1.61	1.85	2.09	2.33	2.43
VPAL																
Female	5	0.99	1.59	2.28	2.91	3.48	4.02	4.53	5.01	5.47	5.91	6.34	6.74	7.14	7.52	7.67
	8	− 1.68	− 1.08	− 0.39	0.24	0.81	1.35	1.86	2.34	2.80	3.24	3.67	4.07	4.47	4.85	5.00
	13	− 3.40	− 2.79	− 2.10	− 1.48	− 0.9	− 0.36	0.15	0.63	1.09	1.53	1.95	2.36	2.75	3.14	3.28
	16	− 3.91	− 3.3	− 2.62	− 1.99	− 1.41	− 0.87	− 0.36	0.12	0.58	1.02	1.44	1.85	2.24	2.62	2.77
	18	− 4.16	− 3.55	− 2.86	− 2.24	− 1.66	− 1.12	− 0.61	− 0.13	0.33	0.77	1.19	1.6	1.99	2.37	2.52
	21	− 4.44	− 3.83	− 3.15	− 2.52	− 1.94	− 1.4	− 0.89	− 0.41	0.05	0.49	0.91	1.32	1.71	2.09	2.24
Male	5	2.09	2.70	3.39	4.01	4.59	5.13	5.64	6.12	6.58	7.02	7.44	7.85	8.24	8.62	8.77
	8	− 0.58	0.03	0.72	1.34	1.92	2.46	2.97	3.45	3.91	4.35	4.77	5.18	5.57	5.95	6.10
	13	− 2.29	− 1.68	− 1.00	− 0.37	0.21	0.75	1.26	1.74	2.20	2.64	3.06	3.47	3.86	4.24	4.39
	16	− 2.80	− 2.2	− 1.51	− 0.88	− 0.31	0.23	0.74	1.23	1.68	2.12	2.55	2.95	3.35	3.73	3.88
	18	− 3.05	− 2.44	− 1.76	− 1.13	− 0.55	− 0.01	0.50	0.98	1.44	1.88	2.30	2.71	3.10	3.48	3.63
	21	− 3.33	− 2.73	− 2.04	− 1.41	− 0.84	− 0.30	0.21	0.70	1.15	1.59	2.02	2.42	2.82	3.20	3.35

*DSF*, Digit Span Forward; *DSB*, Digit Span Backward; *RAVL-I*, Rey’s Auditory Verbal Learning Test-Immediate; *RAVL-D*, Rey’s Auditory Verbal Learning Test-Delayed; *VPAL*, Verbal Paired Associates Learning Test. DSF: adjusted score = raw score + 0.0000017*((Age^3) − 128,919) + 0.472*(Gender − 0.618); DSB: adjusted score = raw score + 0.02*(Age − 43.62); RAVL-I: adjusted score = raw score − 6.72*(log(88-Age) − 3.769) + 106.06*((1/Education) − 0.0697) − 4.214*(Gender − 0.6); RAVL-D: adjusted score = raw score + 0.0481*(Age − 40.8) + 28.678 * ((1/Education) − 0.0697) − 1.635*(Gender − 0.6); VPAL: adjusted score = raw score + 1.357*(sqrt(Age) − 6.514) + 35.603 * ((1/Education) − 0.0733) − 1.106*(Gender − 0.6). Gender was coded as 0 = male and 1 = female

**Table 3:** Equivalent scores for adjusted score of the three tests and relative subtests together with outer (OTL) and inner (ITL) tolerance limits

	Equivalent score							
Test	0	1	2	3	4		OTL	ITL
DSF	≤ 4.02	4.03–5.52	5.53–5.99	6.00–6.19	> 6.19		4.02	4.97
DSB	≤ 2.79	2.80–3.79	3.80–4.57	4.58–4.81	> 4.81		2.79	3.45
RAVL-I	≤ 35.62	35.63–47.19	47.20–51.26	51.27–54.34	> 54.34		35.62	43.79
RAVL-D	≤ 6.24	6.25–9.99	10.00–11.23	11.24–12.15	> 12.15		6.24	8.62
VPAL	≤ 4.02	4.03–5.52	5.53–5.99	6.00–6.19	> 6.19		4.02	4.97

*DSF*, Digit Span Forward; *DSB*, Digit Span Backward; *RAVL-I*, Rey’s Auditory Verbal Learning Test-Immediate; *RAVL-D*, Rey’s Auditory Verbal Learning Test-Delayed; *VPAL*, Verbal Paired Associates Learning Test

**Table 4:** Percentile scoring for adjusted score of the three tests and relative subtests

Percentile	DSF	DSB	RAVL-I	RAVL-D	VPAL
99	9.0	8.5	67.4	15.9	23.3
95	8.2	7.0	65.2	15.0	21.8
90	7.9	6.7	62.6	14.4	20.6
85	7.6	6.1	61.2	13.8	20.2
80	7.5	5.8	60.3	13.5	19.6
75	7.0	5.6	59.7	13.3	19.1
70	7.0	5.6	59.0	13.0	18.6
65	6.8	5.4	57.9	12.8	18.1
60	6.5	5.2	57.1	12.6	17.4
55	6.5	5.1	56.0	12.4	17.2
50	6.2	4.8	54.3	12.1	17.0
45	6.1	4.7	53.3	11.9	16.5
40	6.0	4.6	52.5	11.5	15.9
35	6.0	4.6	51.4	11.2	15.3
30	6.0	4.5	50.6	11.1	14.8
25	5.7	4.3	49.9	10.6	14.3
20	5.6	4.0	48.5	10.2	13.8
15	5.5	3.7	46.4	9.3	12.9
10	5.1	3.6	44.6	8.9	11.8
5	4.5	3.2	42.1	8.4	10.5
4	4.4	3.1	40.9	8.1	10.1
3	4.3	2.9	39.7	7.2	10.0
2	4.0	2.8	36.2	6.4	9.7
1	4.0	2.7	32.5	6.0	8.3

*DSF*, Digit Span Forward; *DSB*, Digit Span Backward; *RAVL-I*, Rey’s Auditory Verbal Learning Test-Immediate; *RAVL-D*, Rey’s Auditory Verbal Learning Test-Delayed; *VPAL*, Verbal Paired Associates Learning Test

### Comparison of neuropsychological tests scores by videoconference and standard setting

In order to compare our defined norms to those already available in the literature, we simulated a sample of 100 aMCI participants. The means and standard deviations of the sample were retrieved from [40] for DSF, DSB, RAVL-I, RAVL-D, and from [41] for VPAL tests. Classification confusion matrices were reported in Table S4 in supplementary materials and performance metrics in Table 5.

**Table 5:** Classification performance metrics between the present and the previous norms

Test	Accuracy	AUC (95% CI)	K (95% CI)	AC1 (95% CI)
DSF	.93	.97 (.94–.99)	.56 (.27–.84)	.92 (.85–.98)
DSB	.70	.82 (.72–.92)	.25 (.07–.44)	.51 (.33–.69)
RAVL-I	.87	.98 (.96–.99)	.68 (.53–.83)	.78 (.66–.90)
RAVL-D	.83	.89 (.82–.96)	.44 (.22–.66)	.76 (.63–.88)
VPAL	.84	.99 (.98–.99)	.23 (.01–.45)	.80 (.70–.91)

*DSF*, Digit Span Forward; *DSB*, Digit Span Backward; *RAVL-I*, Rey’s Auditory Verbal Learning Test-Immediate; *RAVL-D*, Rey’s Auditory Verbal Learning Test-Delayed; *VPAL*, Verbal Paired Associates Learning Test

**Fig. 1:**
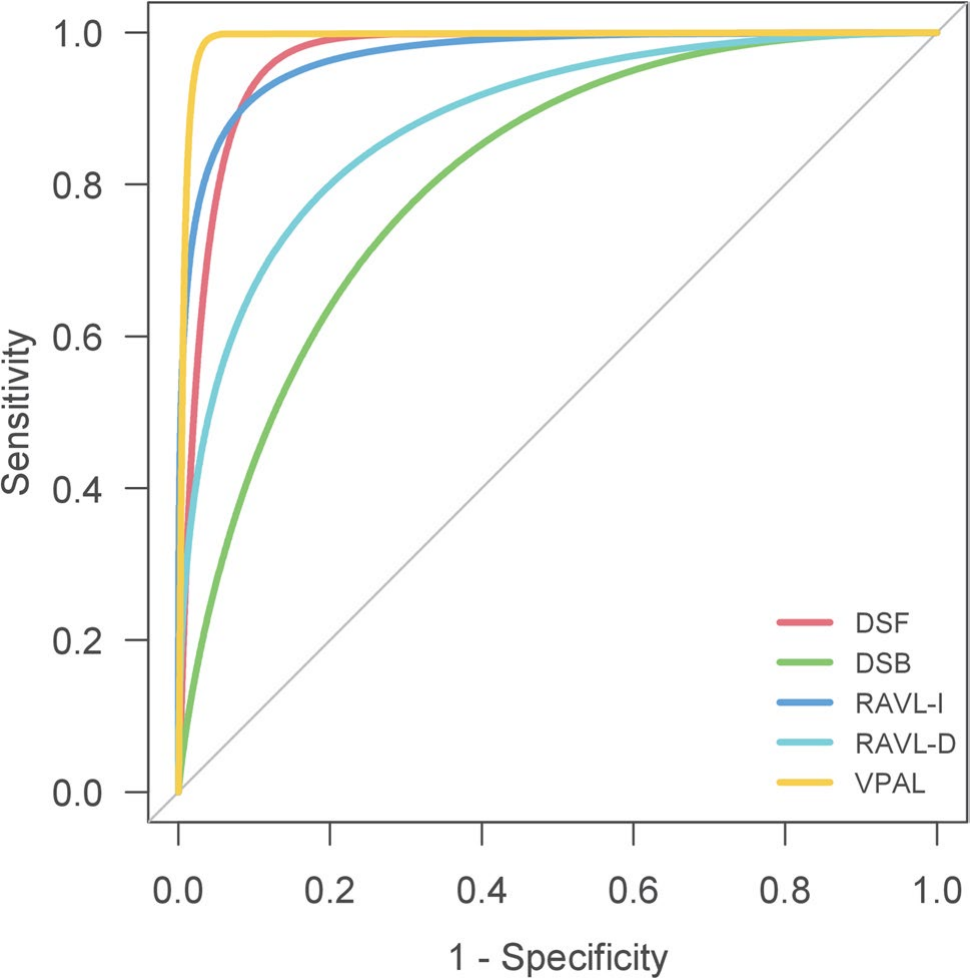
Receiver operating characteristic (ROC) curves for each test. The line of each curve indicates the sensitivity and specificity. For each test, norms from published sources were used to determine reference baseline classification, and norms from the present study were used to predict classification. DSF, Digit Span Forward; DSB, Digit Span Backward; RAVL-I, Rey’s Auditory Verbal Learning Test-Immediate; RAVL-D, Rey’s Auditory Verbal Learning Test-Delayed; VPAL, Verbal Paired Associates Learning Test

Diagnosis concordance established by Cohen’s K showed substantial agreement for RAVL-I, moderate agreement for DSF and RAVL-D, and fair agreement for DSB and VPAL. However, Cohen’s K could return paradoxical results when dealing with unbalanced classification [42]. The AC1 scores demonstrate moderate agreement for DSB, substantial agreement for RAVL-I, RAVL-D, and VPAL, and almost perfect agreement for DSF. AUC values are good for DSB and RAVL-D and optimal for DSF, RAVL-I, and VPAL (Fig. 1). Thus, the diagnostic classification criteria created in our study conform to previous results.

## Discussion

The study aimed to provide normative data of neuropsychological memory tests performed via videoconference. Several studies described videoconferencing assessments as similar to face-to-face settings [6, 18, 21, 24, 44]. The remote setting could expose to many risks and limitations [17, 18], as much as several benefits [10, 13]: (i) covering long distances; (ii) patients with motor deficits can perform a neuropsychological assessment at their place and maintaining continuity in health and care during emergencies.

Therefore, by a well-established procedure, our study defined the new Italian normative data in the videoconferencing assessment of mnemonic performance [38, 39]. Results showed, as expected, that all memory tests correlated to each other and that participants’ scores were influenced mainly by their age and education. Also, results demonstrate the possible use of the Digit Span Forward and Backward, Rey’s Auditory Verbal Learning Test, and the Verbal Paired Associated Learning Test in a remote setting. Scoring system and, hence, its classification are largely comparable to a face-to-face setting. In this new clinical context, our study shows how gender, age, and years of schooling influence the testing and the measurement of memory abilities.

Some recent research pointed out the gender differences in the memory performance. A study on healthy controls, aMCI, and Alzheimer’s disease (AD) patients showed gender differences in verbal memory performance in the Rey Auditory Verbal Learning Test (RAVL) depending on the higher temporal lobe glucose metabolic rates of female participants. Specifically, females’ advantage in verbal memory performance was evident when they showed minimal to moderate temporal hypometabolism rates in aMCI condition to males with similar pathology. The lifelong advantage in verbal memory might represent a form of cognitive reserve that delays verbal memory decline until more advanced pathology stages, as indexed by temporal lobe glucose metabolic rates in females [45]. Females retain this advantage in aMCI despite the reduced hippocampal volume; best memory performance in females delays verbal memory impairment and diagnosis of aMCI and treatment [46].The comparison of the here-defined norms to those already published showed intriguing results. Our new online normative scoring system reports a slight difference in the cut-offs compared with the scoring systems developed in standard settings. As these latter norms refer to studies done several decades earlier, the differences we observed most likely depend on the sample (data collected about 30 years ago) rather than the setting (online vs face-to-face). While we are aware that the comparison of different normative systems with an authentic clinical sample would be the best approach, this was not the primary focus of the current study. Thus, despite its strong explorative nature, we felt that relying on a simulation analysis would have been the best approximation to assess the diagnostic ability of our scoring system and compare it with systems developed in a different setting (i.e., face-to-face). In the future, a specific study involving a direct comparison with new normative data acquired in a face-to-face setting would be useful for answering this emerging question and, also, for better defining other relevant psychometric properties of the online testing (e.g., test–retest and inter-rater reliability). In spite of its limitations, this study provides specific and updated norms for online memory tests.

All in all, cut-off values might be comparable to the scores obtained in face-to-face assessment and already reported in the previous studies. Videoconferencing has proved to be an effective method of diagnosing neurocognitive disorders [1]. In conclusion, the results support the clinical application of videoconferencing modality to assess verbal memory abilities in an Italian sample. Assessing cognitive abilities in a remote setting without in-person support is possible when the platform interface is easy to use, and the participants are healthy or in the early stages of cognitive impairment. These peculiarities make accessible, fast, and handy the use of neuropsychological assessment via videoconference. Also, the verbal nature of memory tests makes them suitable for remote administration. Neuropsychologists can test online and supervise patients over time by employing normative data described in this study for videoconference settings, enhancing diagnostic accuracy [22, 24]. Moreover, this research provides valuable information about how to assess memory abilities in videoconferencing, including how to control potentially problematic environmental factors.

### Remote testing: recommendations

International scientific societies, such as the American Psychological Association, the National Board of Italian Psychology, and the Italian Association of Psychology, published recommendations on using psychodiagnostic tools effectively in online settings. In order to minimise distractions and to maintain patient privacy, testing should take place in an isolated and quiet location following recommendations [47]. Wearing headphones and having adequate lighting should be required while listening to instructions. Participants must look directly into the computer camera and keep their hands close to their chins to prevent note-taking. Some studies have provided recommendations for clinical practice [9], emphasizing the importance of performing a two-step process for patient triage and determining their suitability for assessments in teleneuropsychology. A preliminary video call is required to gather information about a patient’s medical history and determine if the patient has any auditory, visual, or motor deficits that could interfere with the performance of remote tests. Preliminary screening will also determine whether the patient can access suitable assessment equipment (i.e., Internet connection, PC). A final consideration to be made as regards privacy and data protection concerns related to the use of videoconferencing tools. In general, healthcare professionals have the burden to protect the privacy and security of health information and must provide individuals with certain rights with respect to their health information. In the case of online test administration, this burden is extended to the business associate responsible for the video conferencing service used (e.g., Google, Zoom, Microsoft). In order for the data collection and storage to be compliant with the HIPAA, the person responsible for the service (e.g., the healthcare professional or the service administrator in an academic institution) must sign an agreement with the business associate (i.e., the Business Associate Agreement). In this way, the business service pledges to ensure that its platform is safe and secure [48, 49].

## Supplementary Information

ESM 1 (DOCX 11.1 KB)
