# Supplementary material for: Teleneuropsychology: normative data for the assessment of memory in online settings
Source: Neurol Sci. 2022 Oct 5;44(2):529–38. doi: 10.1007/s10072-022-06426-9 (PMC9533275; doi:10.1007/s10072-022-06426-9)
Supplement: Supplementary file 1 — (DOCX 11.1 KB) [file 10072_2022_6426_MOESM1_ESM.docx]

**Supplementary Materials**

**Table S1.** Sample stratification for age. education. and sex (females/males).

|  |  | **Age** | | | | | | | |
| --- | --- | --- | --- | --- | --- | --- | --- | --- | --- |
|  |  | **18-27** | **28-37** | **38-47** | **48-57** | **58-67** | **68-77** | **78-84** | **Total** |
| **Education** | **5-8** | 15/24 | 8/17 | 5/3 | 2/7 | 3/5 | 3/5 | 0/3 | 100 |
|  | **9-13** | 0/0 | 0/0 | 3/0 | 1/1 | 1/2 | 3/7 | 0/3 | 21 |
|  | **14-18** | 6/4 | 2/7 | 3/5 | 5/12 | 3/5 | 5/6 | 0/0 | 63 |
|  | **>18** | 2/3 | 4/6 | 0/1 | 1/1 | 1/0 | 1/0 | 0/0 | 20 |
|  | **Total** | 23/31 | 14/30 | 11/9 | 9/21 | 8/12 | 12/18 | 0/6 | 204 |

**Table S2.** Regression equation for computing adjusted score.

| **Test** | **Regression** | **R^2^** | **Adj R^2^** | **RSE** |
| --- | --- | --- | --- | --- |
| **DSF** | 0.0000017*((Age^3)-128919)+0.472*(Gender-0.618) | .088 | .078 | 1.104 |
| **DSB** | 0.02*(Age-43.62) | .083 | .079 | 1.207 |
| **RAVL-I** | -6.72*(log(88-Age)-3.769)+106.06*((1/Education)- 0.0697)-4.214*(Gender-0.6) | .345 | .334 | 7.44 |
| **RAVL-D** | 0.0481*(Age - 40.8)+28.678 * ((1/Education)-0.0697)-1.635*(Gender-0.6) | .329 | .318 | 2.186 |
| **VPAL** | 1.357*(sqrt(Age) - 6.514)+35.603 * ((1/Education)-0.0733)-1.106*(Gender-0.6) | .363 | .354 | 3.415 |

*Notes.* DSF = Digit span forward; DSB = Digit span backward; RWI = Rey’s auditory verbal learning test  - Immediate; RAVL-D = Rey’s auditory verbal learning test - Delayed; VPAL = Verbal Paired Associates Learning Test. Gender was coded as 1 = female and 0 = male. Adj R^2^ = Adjusted R squared; RSE = Root Square Error.

**Table S3.** Outer and Inner Tolerance Limits

| **Test** | **OTL** | **ITL** |
| --- | --- | --- |
| **DSF** | 4.02 | 4.97 |
| **DSB** | 2.79 | 3.45 |
| **RAVL-I** | 35.62 | 43.79 |
| **RAVL-D** | 6.24 | 8.62 |
| **VPAL** | 4.02 | 4.97 |

*Notes*. DSF = Digit span forward; DSB = Digit span backward; RAVL-I = Rey’s auditory verbal learning test  - Immediate; RAVL-D = Rey’s auditory verbal learning test - Delayed; VPAL = Verbal Paired Associates Learning Test. OTL = outer tolerance limit; ITL = inner tolerance limit.

**Table S4.** Confusion matrices of the classification performance of our and previous tests norms.

| **Test** | **Previous norms** | **Our norms** | | **Total** |
| --- | --- | --- | --- | --- |
|  |  | *Pathological* | *Normal* |  |
| **DSF** | *Pathological* | 5 | 7 | 12 |
|  | *Normal* | 0 | 88 | 88 |
|  | **Total** | 5 | 95 | 100 |
|  |  | *Pathological* | *Normal* |  |
| **DSB** | *Pathological* | 11 | 6 | 17 |
|  | *Normal* | 24 | 59 | 83 |
|  | **Total** | 35 | 65 | 100 |
|  |  | *Pathological* | *Normal* |  |
| **RAVL-I** | *Pathological* | 66 | 0 | 66 |
|  | *Normal* | 13 | 21 | 34 |
|  | *Total* | 79 | 21 | 100 |
|  |  | *Pathological* | *Normal* |  |
| **RAVL-D** | *Pathological* | 73 | 5 | 78 |
|  | *Normal* | 12 | 10 | 22 |
|  | **Total** | 85 | 15 | 100 |
|  |  | *Pathological* | *Normal* |  |
| **VPAL** | *Pathological* | 3 | 16 | 19 |
|  | *Normal* | 0 | 81 | 81 |
|  | **Total** | 3 | 97 | 100 |

*Notes.* DSF = Digit span forward; DSB = Digit span backward; RWI = Rey’s auditory verbal learning test  - Immediate; RAVL-D = Rey’s auditory verbal learning test - Delayed; VPAL = Verbal Paired Associates Learning Test.
